# Argumentation Based Joint Learning: A Novel Ensemble Learning Approach

**DOI:** 10.1371/journal.pone.0127281

**Published:** 2015-05-12

**Authors:** Junyi Xu, Li Yao, Le Li

**Affiliations:** Science and Technology on Information System and Engineering Laboratory, National University of Defense Technology, Changsha, Hunan, P.R. China; Leibniz Institute for Age Research, GERMANY

## Abstract

Recently, ensemble learning methods have been widely used to improve classification performance in machine learning. In this paper, we present a novel ensemble learning method: argumentation based multi-agent joint learning (AMAJL), which integrates ideas from multi-agent argumentation, ensemble learning, and association rule mining. In AMAJL, argumentation technology is introduced as an ensemble strategy to integrate multiple base classifiers and generate a high performance ensemble classifier. We design an argumentation framework named Arena as a communication platform for knowledge integration. Through argumentation based joint learning, high quality individual knowledge can be extracted, and thus a refined global knowledge base can be generated and used independently for classification. We perform numerous experiments on multiple public datasets using AMAJL and other benchmark methods. The results demonstrate that our method can effectively extract high quality knowledge for ensemble classifier and improve the performance of classification.

## Introduction

Nowadays ensemble methods represent one of the main current research lines in machine learning [1, 2] and their application range over a large number of problems such as distributed text classification, time series clustering on network and pedestrian detection [3–6]. Ensemble methods [7, 8] are designed to increase the accuracy of a single classifier by training several different classifiers and combining their decisions (by voting or weighted voting) to output a single class label, such as Bagging [9], Boosting [10] and Random Forest [11]. Numerous empirical and theoretical studies have demonstrated that ensemble classifier can always attain higher accuracy than single classifier [12].

However, traditional ensemble learning methods have some drawbacks. 1) From the perspective of integration manner, traditional ensemble methods mostly use weighted voting (such as Bagging) to integrate multiple base classifiers. While this “majority-voting” approach is relatively too simple and lack of the interpretability for users. 2) From the perspective of integration object, ensemble methods only integrate classification results of base classifiers rather than internal classification knowledge in base classifiers. 3) From the perspective of integration result, traditional ensemble classifier consists of all base classifiers which are essential in the classification process. Therefore, ensemble classifier cannot be independent of base classifiers, which leads to high classification cost. In order to overcome the weaknesses of traditional ensemble learning methods, we introduce a new ensemble strategy based on multi-agent argumentation.

With the development of artificial intelligence, argumentation technology as a new way of multi-agent interaction can imitate human decision-making process to realize the conflict resolution and knowledge integration, which takes advantage of group intelligence for problem solving. In recent years, argumentation with data mining technology has been extensively studied, such as argumentation in association rule mining [13–17], machine learning based on argumentation [18, 19], argumentation with inductive learning and case-based reasoning [20–22], argumentation in reinforcement learning [23, 24].

Due to the above advantages of argumentation, this paper proposes a novel ensemble learning approach: argumentation based joint learning, which introduces multi-agent argumentation technology into the field of ensemble learning. In our method, agents perform data mining on respective datasets to construct base classifiers independently at first. Then, base classifiers evaluate their individual knowledge by using argumentation. Finally, high quality global knowledge can be extracted to generate ensemble classifier.

Argumentation based joint learning method has the following advantages. 1) The ensemble strategy of argumentation is more logical and explicable than voting. This is because argumentation can make use of classification knowledge as arguments to realize the strict dialectic analysis process. 2) Argumentation technology can evaluate the knowledge of base classifiers, and automatically extract high-quality knowledge to form the global knowledge base for classification, which achieves the integration of classification knowledge, rather than a simple combination of classification results. 3) The global knowledge base of ensemble classifier can be directly applied to solve the classification problem, and base classifiers are unnecessary in the final classification process, which can greatly improve the efficiency of classification.

The article is organized as follows. Section 2 introduces the related research works about ensemble learning and argumentation; Section 3 presents the principles of argumentation based joint learning model; then realization process of the model and algorithm will be given in Section 4; Section 5 introduces the argumentation platform Arena used in joint learning model. Experimental results on public datasets are presented and discussed in section 6 and 7. Finally section 8 summaries the contributions and future works of this paper.

## Related Works

Recent years, ensemble learning and argumentation technology both attract much attention in the field of data mining. In this section, we will focus on the related works about ensemble learning and argumentation in data mining.

### Ensemble learning

Research in many areas has shown the advantages of ensemble methods both theoretically and empirically [25, 26]. In ensemble methods, learners composing an ensemble are usually called base learners. Methods used to generate base learners can be divided into following types: resampling of the training set (such as Bagging [9], Boosting [10]), manipulation of input variables (such as Random Subspaces [27] and Rotation Forests [28]), manipulating the output targets (such as error correcting output code ensemble method [29]), and introducing randomness (such as Random Forest [11]).

When forming ensembles, creating diverse classifiers (but maintaining their consistency with the training set) is a key factor to make them accurate. The research in [12] makes a distinction between two types of ensemble learning algorithms, those which encourage diversity (making classifiers showing a great deal of variety to avoid over-fitting) implicitly, and those which en-courage it explicitly.

The vast majority of ensemble methods are implicit, in that they provide different random subsets of the training data to each learner. The random differences between the datasets might be in the selection of examples (the Bagging algorithm), the selection of features (Random Sub-spaces [27] or Rotation Forests [28]), or combinations of the two (the Random Forests [11] algorithm). An alternative is to explicitly encourage diversity, constructing each ensemble member with some measurement ensuring it is substantially different from the other members, such as constructing base learners on weighted versions of the training set (Boosting [26, 30]), explicitly altering the distribution of class labels (DECORATE algorithm [31]), explicitly managing the accuracy-diversity tradeoff (Negative Correlation Learning [32, 33]).

### Argumentation in data mining

Recent years, lots of researches focus on argumentation theory as knowledge integration method in data mining domain. A number of approaches have been proposed to integrate argumentation and machine learning. Governatori and Stranieri investigate the feasibility of KDD in order to facilitate the discovery of defeasible rules for legal decision making [17]. In particular they argue in favor of Defeasible Logic as an appropriate formal system in which the extracted principles should be encoded in the context of obtaining defeasible rules by means of induction-based techniques.

The idea that argumentation might be useful for machine learning was discussed in [19], since argumentation could provide a sound formalization for both expressing and reasoning with uncertain and incomplete information. Since the possible hypotheses induced from data could be considered an argument, and then by defining a proper attack and defeat relation, a sound hypotheses can be found.

Onta*ñó*n and Plaza in [21] research concept learning, and put forward a multi-agent inductive learning framework A-MAIL, which integrates inductive learning, case-based reasoning and argumentation theory. In this framework, multi-agent Inductive Learning consists of three stages: individual induction; argumentation process; and belief revision. The proposed method is different from ours. In A-MAIL, each agent just use argumentation based inductive learning to revise their own knowledge and multi-agent system do not form a global knowledge base. Moreover, A-MAIL focuses on inductive learning while our method focuses on association rules.

Wardeh proposes argumentation from experience in [13], and combines argumentation theory with data mining techniques. Agent gets association rules as their arguments in the library of their own experience through data mining. PADUA argumentation model is designed to achieve two party argumentation processes and resolve uncertainties classification problems. Later, PISA model is designed in [14] in order to solve the multi-classification problem. However, PISA has complicated strategy and complex argumentation process, so that the model does not have general applicability. Subsequently, the concept of collaborative group of Agents is proposed for arguing from experience in [15].

In order to enhance the versatility of PISA, Wardeh simplifies the speech acts and removes a complex strategy in argumentation in [16]. The improved model can be used to solve the following problem in classification: multi-agent classification, ordinal classification and imbalance classification. Although the simplified model improves the versatility, its classification accuracy is decreased.

In this paper, argumentation based joint learning is different from PISA model. PISA model focuses on classification problem and its goal is to improve the classification accuracy through multi-agent argumentation, while the purpose of our method is to realize multi-agent joint learning and knowledge extraction from data mining. Argumentation in PISA is driven by the classification result while our method is driven by classification knowledge.

## Argumentation based multi-agent Joint Learning

**Fig 1:**
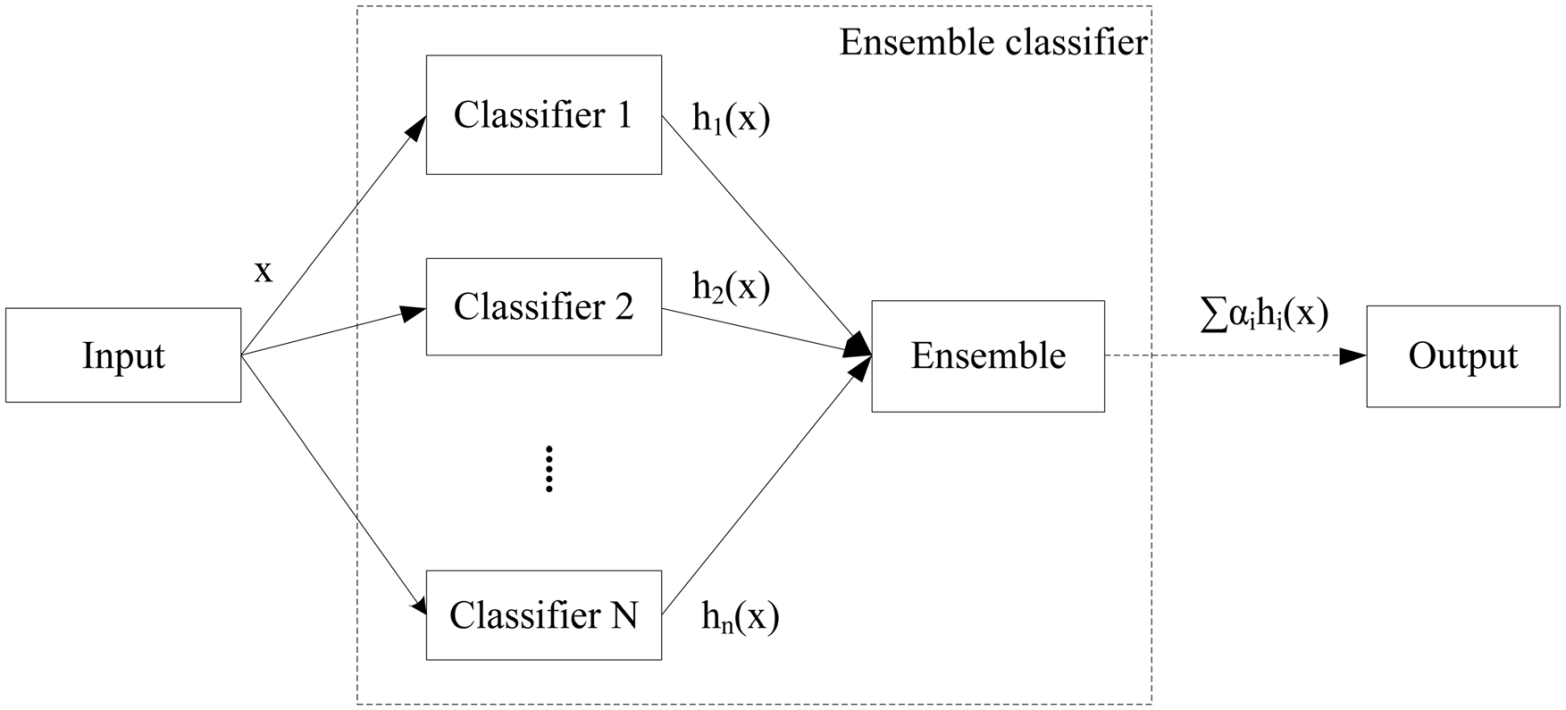
Ensemble learning.

**Fig 2:**
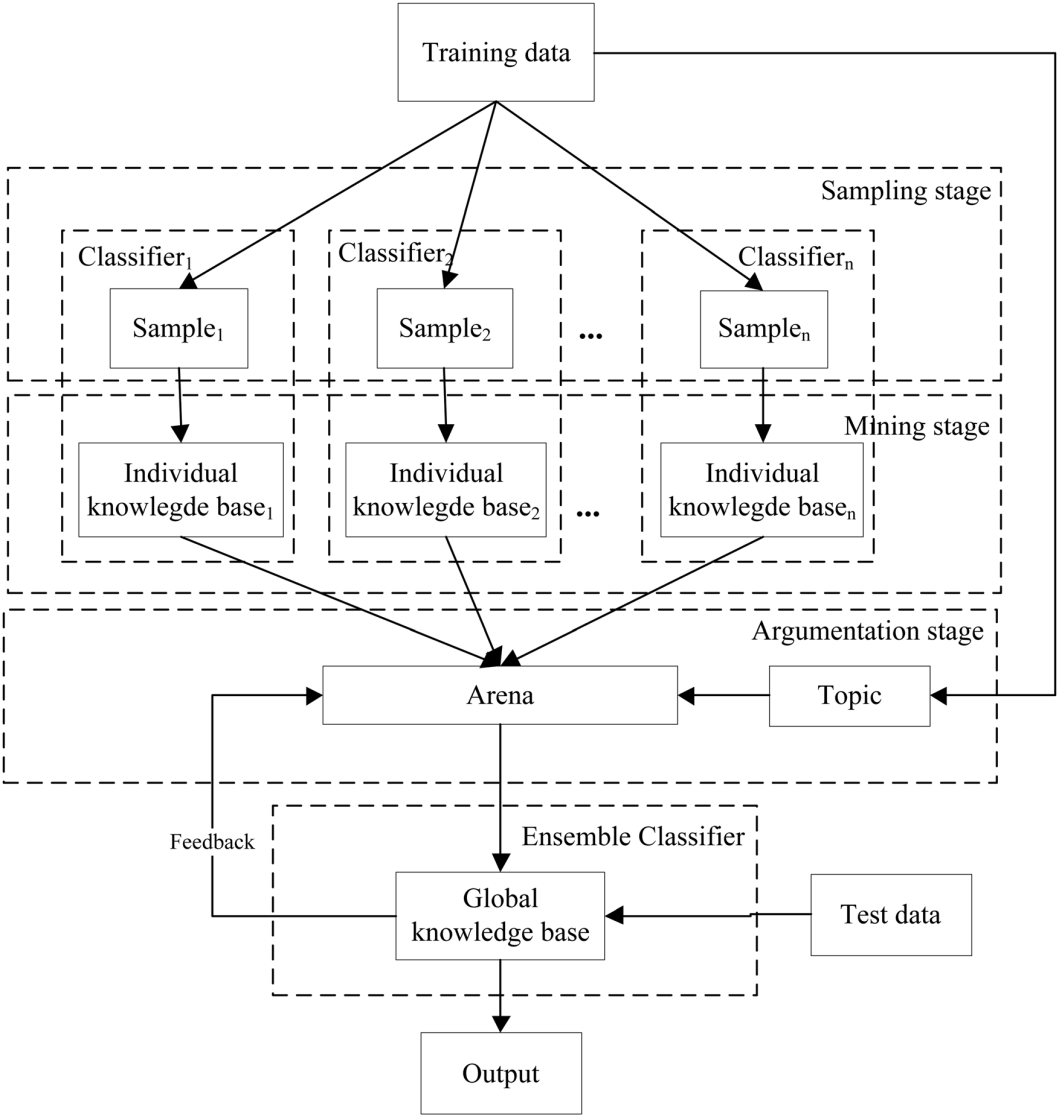
Argumentation based joint learning.

In this paper, we propose the argumentation based multi-agent joint learning method AMAJL, to achieve ensemble learning of multiple classifiers. Similar to the idea of ensemble learning (shown in Fig 1), joint learning method uses the idea of integrating multiple base classifiers to get better ensemble classifier as well. The model of AMAJL is shown in Fig 2. From the following figures, we can illustrate the integration of base classifiers and the generation of ensemble classifier in our method, which are quite different from traditional ensemble methods.

From Fig 2 we can see, argumentation based joint learning method consists of three stages. First in sampling stage, individual agent generates its training sample by sampling with replacement from the training data. Next in mining stage, agents perform data mining on respective samples to generate individual knowledge bases. Then in argumentation stage, for each sample (can be treated as *argumentation topic*) in training dataset, agents use individual knowledge to perform argumentation on Arena for obtaining an optimal knowledge of this topic, which will be stored in the global knowledge base of ensemble classifier. Meanwhile, global knowledge in ensemble classifier can give a feedback to guide the subsequent joint learning process. With the increase of input topics, global knowledge base will keep growing until it converges. In classification process, ensemble classifier can be used directly to classify the test data.

In the above process, argumentation technology plays a crucial role in joint learning method. By using argumentation technology, joint learning model can evaluate the individual knowledge, eliminate low-quality knowledge and extract the optimal classification knowledge to construct high performance ensemble classifier.

With the idea of swarm intelligence (intelligent behavior manifested by group cooperation), our method focuses on knowledge integration and extraction by multi-agent argumentation which is not only more similar to human decision-making process, but also easier for users to understand than traditional ensemble methods.

## Process of AMAJL

Argumentation based joint learning can be divided into two processes: base classifier generation and argumentation based integration. In base classifier generation process, agents use sampling with replacement to get different training sets, and then data mining technology is used to generate base classifiers. In integration process, multiple base classifiers use argumentation technology to generate ensemble classifier.

### Base classifier generation

In this paper, we focus on data variation-based ensembles [34], which consist in the manipulation of the training examples in such a way that each classifier is trained with a different training set. Sampling with replacement is used to construct training sample *S*_*i*_ for individual *agent*_*i*_ from the original training data set *D*. The size of training sample *S*_*i*_ of each agent is same with the original training set *D*. However, due to the sampling with replacement, training samples for each classifier are different, and there may be duplicate cases in individual sample *S*_*i*_.

In traditional ensemble learning, base classifier must be able to classify. However, in our method, base classifier is used to generate classification knowledge rather than classification result. Therefore, lots of classification algorithms (such as Support Vector Machine [35]), which cannot generate classification knowledge, are inapplicable in our base classifier generation.

Therefore, we choose association rule mining algorithm to generate base classifiers. This is because association rule can effectively represent not only the individual knowledge of agents but also the argument for agents to communicate in argumentation process. In addition, association rule can be directly used in classification as well.

In this process, each agent performs association rule mining using Apriori-TFP algorithm [36] on local sample *S*_*i*_ to get association rules exceeding certain support and confidence threshold. In addition, we not only record association rules, but also record application condition as well as exception cases of each association rules. Application condition means the necessary condition that all the cases must satisfy when the conclusion of association rule is true. In other word, if an attribute is always accompanied with the appearance of investigated conclusion of the association rule in every case, we can infer that the attribute is application condition of the conclusion. Exception case means the cases in sample that contains the premises of the rule but not contains the conclusion.

Through the above process, each agent can get a set of extended association rules (*AR*), which can be treated as individual knowledge of base classifier. An element of *AR* is defined as follows: Conclusion: *w*; Premises: *l*_1_, *l*_2_, …, *l*_*n*_; Confidence: *c*; Conditions:*u*_1_, *u*_2_, …, *u*_*s*_;¬*v*_1_, ¬*v*_2_, …, ¬*v*_*t*_; Exceptions:*e*_1_, …, *e*_*k*_.

When a case comes, instead of predicting the class label, base classifier uses the rule, which matches the case and has the highest confidence, to argue with other base classifiers for extracting the optimal knowledge of the current case.

By association rule mining, agents can extract potential individual knowledge to realize the externalization of tacit knowledge, which provides support to the subsequent argumentation process.

### Argumentation based integration

Ensemble methods requires a mechanism to combine base classifiers for generating ensemble classifier, therefore in this process we introduce argumentation technology to achieve the integration of base classifiers.

From knowledge spiral model [37] in the field of knowledge management, we can find that organizational knowledge is extracted from the common knowledge among individuals through communication. Inspired by the above knowledge extraction process, in our model, high-quality knowledge of ensemble classifier is extracted from base classifiers by argumentation.

In the process of argumentation based integration, base classifiers perform argumentation on Arena to integrate individual knowledge. In the beginning of argumentation, for a specific topic *t* ∈ *D*, each base classifier *A*_*i*_ use rules in knowledge base *AR*_*i*_ as arguments to express their opinions, which can exchange and evaluate their local knowledge *AR*_*i*_ with other classifiers. After sufficient argumentation, base classifiers can reach an agreement and obtain a winning rule *k*_*t*_ which is the optimal classification knowledge for current topic *t* and stored in the global knowledge base *K* of ensemble classifier. With lots of training cases in *D*, high-quality knowledge in global knowledge base is accumulating constantly and eventually converges to a stable set of rules. Through the accumulation of high-quality knowledge, high-performance ensemble classifier can be eventually generated.

In addition, the global knowledge base also gives a feedback on the argumentation process. Under the two conditions, winning rules *k*_*t*_ will not be added to the global knowledge base *K*. The first condition is that the winning rule *k*_*t*_ extracted by argumentation has already existed in the global knowledge base *K*(∃*k* ∈ *K*∧*k*_*t*_ = *k*); and the second is classification rules of the global knowledge base *K* can better match the current topic *t* than the winning rule *k*_*t*_ (∃*k* ∈ *K*∧*premises*(*k*) ⊂ *t*∧*length*(*k*) > *length*(*k*_*t*_), where *premises*(*k*) is the set of premises of rule *k*, and *length*(*k*) is the length of premises of rule *k*).

The global knowledge base *K* in ensemble classifier is composed of extended association rules, which can be used for prediction independently. When a new (unknown) case *x* comes, ensemble classifier selects the rule *k* which matches the case *x* (*k* ∈ *K*∧*premises*(*k*) ⊂ *x*) and has the highest confidence (*conf*(*k*) = max{*conf*(*k*′)∣*k*′ ∈ *K*}) to predict the class label of the case *x*.

### Algorithm

The main control algorithm of argumentation based joint learning model is in Table 1.

**Table 1:** Algorithm.

**Algorithm** MainControl of AMAJL
**Input**: Training Set *D*; Agent *A*_*i*_; min_sup *ms*; min_conf *mc*;
Initial global knowledge base *K*
1: **For** each (*A*_*i*_) **do**
2: *S*_*i*_ = Sampling_with_replacement (*D*);
3: *AR*_*i*_ = Associasion_Rule_Mining (*S*_*i*_, *ms*, *mc*);
4: **End for**
5: **While** (*t* ∈ *D*) **do** // *t* is the topic of argumentation
6: *k* = Arena(*t*, *A*_*i*_, *AR*_*i*_) //argumentation on Arena
7: { Broadcast *t*;
8: *Q*_*p*_ = Apply_For_Participant (*A*_*i*_); // *Q*_*p*_ is the queue of participants
9: Participant *P*_*i*_ = Get_Participant (*Q*_*p*_);
10: **For** each *P*_*i*_ **do**
11: Propose_Argument (*P*_*i*_, *t*, *AR*_*i*_);
12: **End for**
13: **If** *P*_*i*_ == silence **then**
14: select next participant *P*_(_ *i*+1);
15: **End if**
16: **If** only *P*_*i*_ == active **then**
17: *P*_*i*_ = winner;
18: *k*_*t*_ = Main_Argument(*P*_*i*_);
19: Return (*k*_*t*_);
20: **End if**
21: } // The argumentation is over
22: **if** *k*_*t*_ ∉ *K* and *k*_*t*_ is better than ∀*k* ∈ *K* for *t* **then**
23: *K* = Add_To_Knowledge_Base(*k*_*t*_);
24: **End if**
25: **End while**
26: Return (*K*); //Get the global knowledge base
**Output**: Global knowledge base *K*

## Argumentation Framework: Arena

In argumentation based joint learning method, multi-agent argumentation is implemented on the Arena model. This section will describe the basic structure of Arena, arguments, speech acts and terminate conditions of argumentation.

### Basic structure of Arena

**Fig 3:**
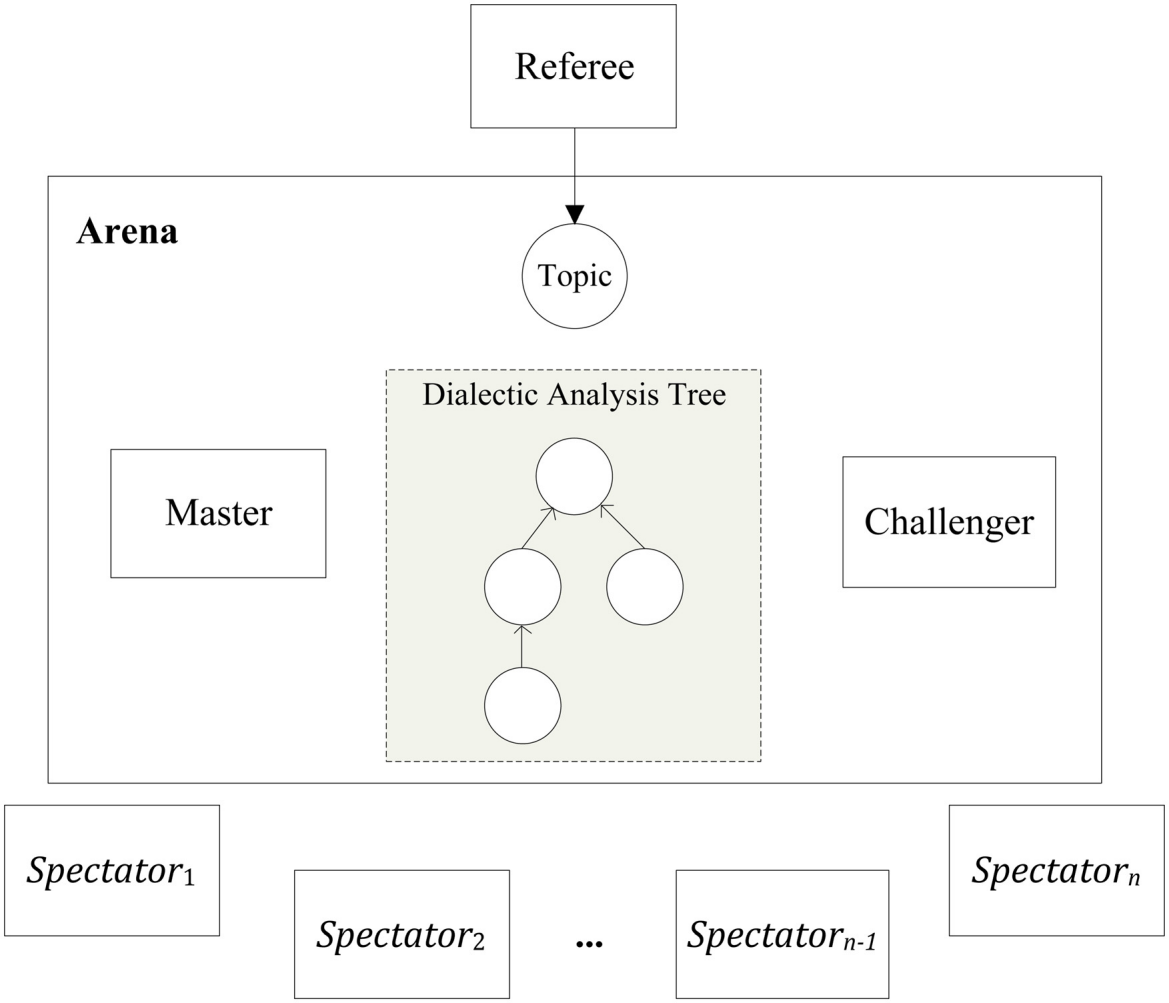
The basic structure of Arena model.

Arena is a dialectic analysis model for multiparty argument games. In Arena, we designed four roles: Referee, Master, Challenger and Spectator. The whole process of argumentation is stored in the dialectic analysis trees. The basic structure of Arena model is shown in Fig 3.

In Arena, Referee doesn’t participate in argumentation but manages the argumentation process according to dialogue rules of Arena. All the participating agents will play a role of Master, Challenger and Spectator. During an argumentation, participating agents need to compete for Master or Challenger continually. And there can be only one Master and one Challenger to take part in the argumentation, while other participants are not allowed to speak when they are just Spectators.

At the beginning of an argumentation, Referee broadcasts the discussion topic, and the first agent who proposes its opinion about the current topic will be selected as the Master of Arena. All the other participants whose options are different from Master can challenge the Master and form the queue of challengers, and the first participant in the queue is selected to be the Challenger of Arena. All the other participant agents except Master and Challenger are Spectators of Arena.

Once Master and Challenger are identified, agents can use the speech acts for constructing Master-Challenger dialogues in Arena.

### Argument schema

In argumentation, for each topic under discussion, agents can produce arguments from their individual knowledge base *AR*.

Suppose that *x* represents a topic under discussion. Argument schema is defined as follows: {Premises: *l*_1_(*x*), *l*_2_(*x*), …, *l*_*n*_(*x*) → Conclusion: *w*(*x*); Confidence: *c*; Exceptions:*e*_1_, …, *e*_*k*_; Conditions: *u*_1_(*x*), *u*_2_(*x*), …, *u*_*s*_(*x*); ¬*v*_1_(*x*), ¬*v*_2_(*x*), …, ¬*v*_*t*_(*x*)}.

Such argument schema for experience can be read as follows: In my experience, if anything *x* doesn’t belong to {*e*_1_, …, *e*_*k*_}, with features *u*_1_, *u*_2_, …, *u*_*s*_ and not with features *v*_1_, *v*_2_, …, *v*_*t*_, then *x* with features *l*_1_, *l*_2_, …, *l*_*n*_, are *W* with probability *c*.

### Speech acts

Speech act is the language of participant to communicate with others in argumentation. In Arena, there are six speech acts: **ProposeOpinion**, **DistinguishRule**, **CounterRule**, **BeInapplicable**, **BeAnException**, and **Defeated**.

**ProposeOpinion**: agent *A*_*i*_ uses this speech act to propose the opinion about the topic *t* by selecting the rule *k* which matches the topic *t* (*k* ∈ *AR*_*i*_∧*premises*(*k*) ⊂ *t*) and has the highest confidence from his local knowledge *AR*_*i*_ (*conf*(*k*) = max{*conf*(*k*′)∣*k*′ ∈ *AR*_*i*_}).

**CounterRule**: agent *A*_*i*_ uses this speech act to attack the adversary’s argument *k*′ by selecting the rule *k* with higher confidence (*k* ∈ *AR*_*i*_∧*premises*(*k*) ⊂ *t*∧*conf*(*k*) > *conf*(*k*′)).

**DistinguishRule**: agent *A*_*i*_ uses this speech act to attack the adversary’s argument *k*′, and allows the addition of some new premise(s) to a previously proposed rule *k*′, so that the confidence of the new rule *k* is lower than *k*′ (*k* ∈ *AR*_*i*_∧*premises*(*k*) ⊂ *t*∧*premises*(*k*) ⊃ *premises*(*k*′)∧*conf*(*k*) < *conf*(*k*′)). It means the adversary’s argument *k*′ is unreasonable because of not considering enough information about the topic *t*.

**BeInapplicable**: agent *A*_*i*_ uses this speech act to state that the adversary’s argument *k*′ is inapplicable to this topic *t*, since the Conditions {*u*_1_, *u*_2_, …, *u*_*s*_;¬*v*_1_, ¬*v*_2_, …, ¬*v*_*t*_} of *k*′ in his own knowledge base *AR*_*i*_ doesn’t satisfy topic *t* (({*u*_1_, *u*_2_, …, *u*_*s*_} ⊄ *t*)∨(*t*∩{*v*_1_, *v*_2_, …, *v*_*t*_} ≠ ∅)), which means agents have different experience for *k*′.

**BeAnException**: agent *A*_*i*_ uses this speech act to state that the topic *t* is in exception {*e*_1_, …, *e*_*k*_} of adversary’s argument *k*′ with the same Premises and Conclusion in his own knowledge base *AR*_*i*_ (*t* ∈ {*e*_1_, …, *e*_*k*_}).

**Defeated**: agent *A*_*i*_ uses this speech act to state that he is defeated.

In Arena, each agent only uses these speech acts from his knowledge base to attack the adversary according to Table 2.

**Table 2:** Speech acts in Arena.

Move	Label	Next Move	Attack with New Rule
1	ProposeOpinion	2,3,4,5	yes
2	CounterRule	3,4,5	yes
3	DistinguishRule	4,5	yes
4	BeInapplicable	0	no
5	BeAnException	0	no

### Termination condition

If Master is defeated by Challenger, this Challenger will become the new Master, and he can propose his opinion about the current topic from his own knowledge base. All the other participants decide whether or not to challenge this new opinion. Noted that the defeated argument of the old Master can’t be used again, the old Master can only produce a new argument to apply for Master once more.

Otherwise, if Challenger is defeated, the next participant in the challenger queue will be selected as the new Challenger, and the argumentation continues.

Termination conditions of Arena are as follows: if Master can defeat all the challengers, Master wins the argumentation and Master’s winning rule will be considered as the optimal knowledge for the current topic; if there is no agent applying for Master, the argumentation is tie. Since the number of arguments produced by participants is finite and the defeated arguments can’t be allowed to use repeatedly, the termination of argumentation can be guaranteed.

## Experimental Setup

In this section we will first introduce data sets collected for experiment and representative baseline methods for comparison. Then we will describe the details of experimental methodology.

### Datasets

In order to empirically evaluate AMAJL we use seven public machine learning datasets in Table 3 from the UCI Machine Learning Repository.

**Table 3:** Datasets.

Name	Number of Attributes	Number of Instances	Number of Items	Missing Values?	Support	Confidence
Breast-Cancer	9	286	43	Yes	5%	60%
Lymph	18	148	63	No	25%	60%
Voting	16	435	34	Yes	35%	70%
Spect Heart	22	267	46	No	30%	60%
Nursery	8	12960	31	No	1%	50%
Scale	4	625	23	No	2%	50%
Tic-Tac-Toe	9	958	29	No	2%	50%

In Table 3, two datasets contain missing values. In order to avoid impact of missing values on experiment results, we remove the instances with missing values in *breast-cancer* and *voting*. In this experiment, we use the confidence and support threshold in Table 3 to mine association rules on datasets. Confidence and support threshold are selected according to dimensions of different datasets. For datasets with higher dimension, we set a relative higher support threshold value, and vice versa.

Item is a common term in association rule mining to represent the attribute value of instances. The association rule model represents rules where some set of items is associated to another set of items. Therefore, the number of items has direct impact on the complexity of association rule mining.

In our model, the computation complexity of base classifier generation is largely dependent on the complexity of the underlying association rule mining algorithm. The computation complexity of argumentation is dependent on the complexity of finding rules matching the instances in knowledge base. We assume that the number of base classifiers is *NC*, the average number of rules in knowledge bases is *NR*, and the number of attributes is *NA*, then computation complexity of argumentation is *O*(*NC* × *NR* × *NA* ^2^). As can be seen, the dimension of datasets directly affects the computation complexity of argumentation. With the dimension increases, the efficiency of argumentation will be reduced.

### Baseline methods

In order to validate the performance of our method AMAJL, we will use three ensemble learning methods Bagging [38, 39], AdaBoost [40, 41] and Random Forest [11] to observe the pros and cons of different ensemble methods. In addition, we compared AMAJL with respect to the common single classifiers: centralized association rule classification algorithm TFPC [42], SVM [43] (linear, radial), NaiveBayes [44], CART [45], k-NN [46].

All these baseline methods except TFPC are implemented on the data mining software WEKA [47], and base classifiers of Bagging and AdaBoost choose the CBA algorithm (Classification Based on Associations) [48].

In the experiment, we use four base classifiers for AMAJL, Bagging and AdaBoost, and choose 100 trees for Random Forest. In addition, baseline methods based on association rule (TFPC, Bagging and AdaBoost) use the same confidence and support threshold in Table 3. The resting baseline methods choose the default parameters in WEKA.

### Experimental Methodology

In order to get more accurate results in the experiment, we apply “Ten-fold Cross Validation” (TCV) tests to evaluate the performance of different methods. In the experiment, we use Accuracy and Rule Number as the evaluation measures to compare the classification performance of different methods.

Accuracy is used to measure the classification ability of classifier, which is calculated as the number of cases classifier correctly predicted.

Rule Number represents the scale of classification knowledge in classifier which is used to evaluate the ability for knowledge extraction. In our experiment, rule number of AMAJL is the number of association rules in the global knowledge base of ensemble classifier. While rule number of TFPC method is the number of classification rules contained in the single classifier. As to Bagging and AdaBoost, multiple base classifiers need to be involved in classification, so the rule number results of Bagging and AdaBoost correspond to the average of the rule number obtained by each base classifier.

## Experimental Results and Discussion

In this section we first give a specific case to illustrate the argumentation process on Arena, and then describe the knowledge accumulation and convergence process in AMAJL model. Next we evaluate the classification performance of AMAJL method compared with other baseline methods, and finally give analysis of experimental parameters.

### Case study

In order to better illustrate the argumentation process, we use a specific case in *Nursery* dataset to show the multi-agent argumentation process. *Nursery* dataset was derived from a hierarchical decision model originally developed to rank applications for nursery schools. It was used during several years in 1980’s when there was excessive enrollment to these schools in Ljubljana, Slovenia, and the rejected applications frequently needed an objective explanation. In this experiment, we used 4 agent involved in the argumentation.

Given a case as the topic, it should be classified as “special priory”. The case has attributes: the parents have a usual occupation, has critical nursery, complete family, 2 children, critical housing, inconvenient finance, problematic social conditions and priority health conditions.

After the Referee broadcasts this discussion topic, each agent produces his opinion for the case and applies for Master.

*Agent*_2_ becomes the first Master, and proposes his opinion as follows:

“According to my experience, this case should be ‘special priory’ based on the rule: *R*_1_ = {critical_nursery, critical_housing, priory_health → spec_prior; 97.67%; *E*_1_; ∅; not_recom_health.}”

*Agent*_4_ challenges *Agent*_2_ by using *DistinguishRule* as follows:

“According to my experience, *R*_1_ is unreasonable. Because *R*_2_ = { critical_nursery, critical_housing, inconvenient_finance,priory_health → spec_prior; 94.28%; *E*_2_; ∅;not_recom_health }, *R*_2_ add the premise ‘inconvenient_finance’ to *R*_1_, while the confidence drops 3.39%.”

*Agent*_2_ cannot propose any rule to attack *Agent*_4_ and concedes that he is defeated.

Then *Agent*_4_ becomes the new master, and proposes his own opinion. Then argumentation process continues to move forward.

**Fig 4:**
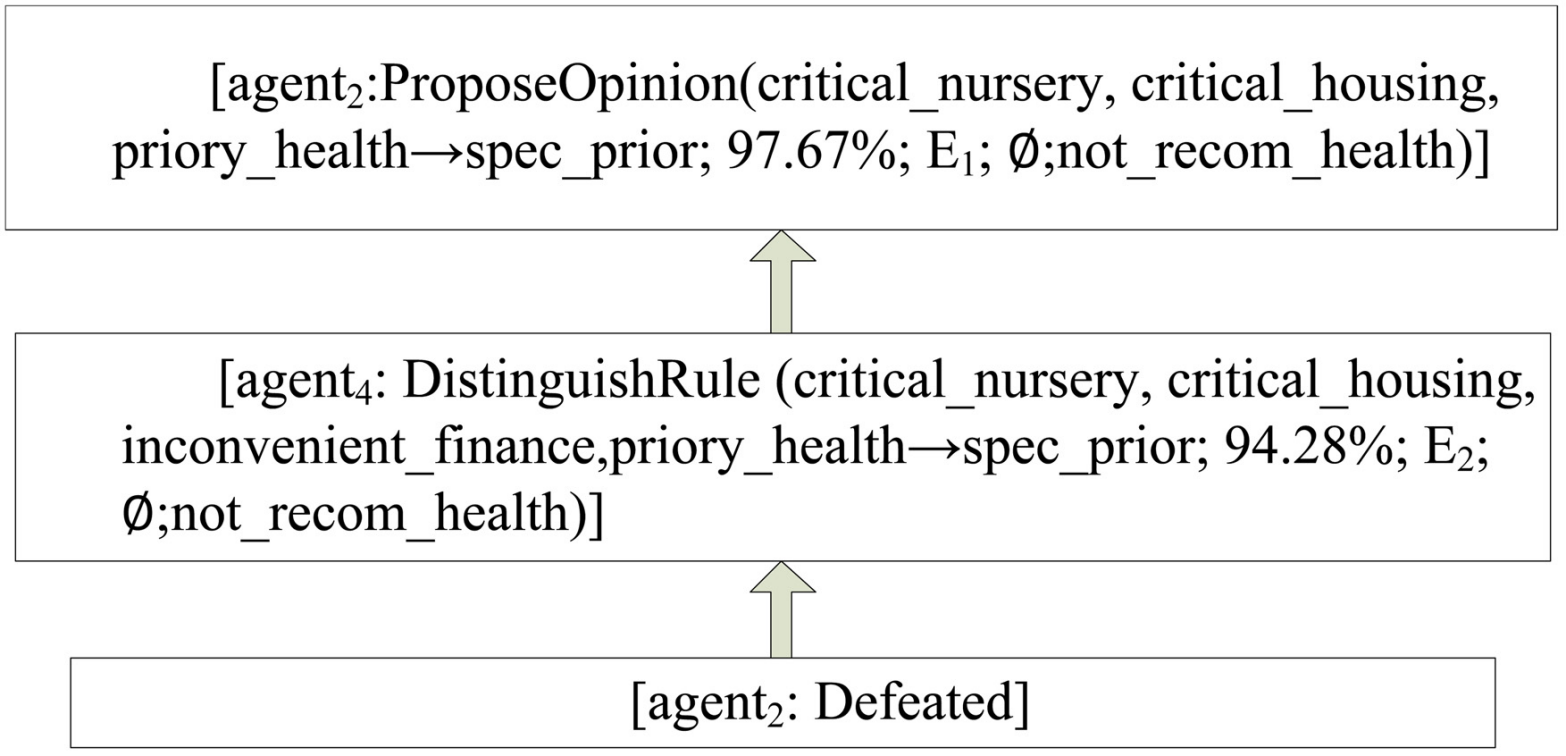
The dialectic analysis tree of Master_1_.

**Fig 5:**
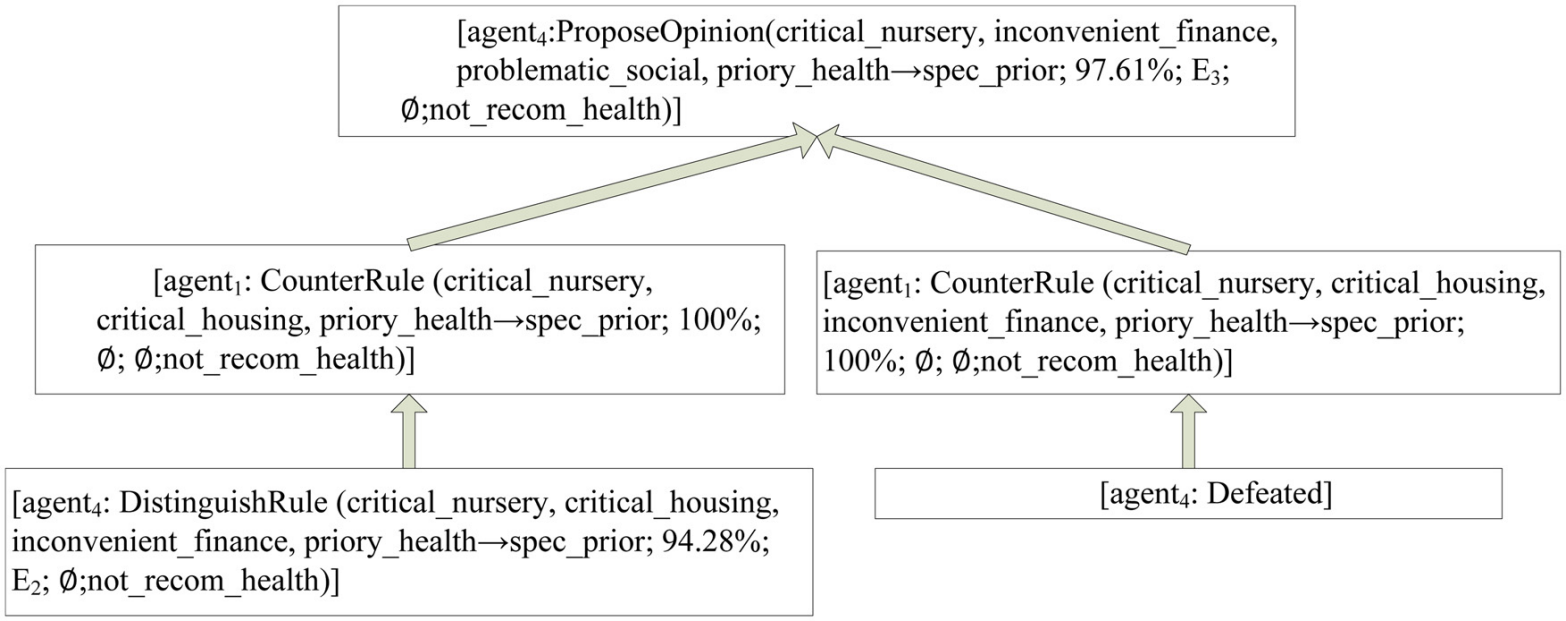
The dialectic analysis tree of Master_2_.

**Fig 6:**
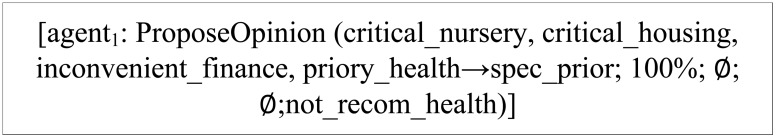
The dialectic analysis tree of Master_3_.

The dialectic analysis tree of the whole argumentation process is depicted on Figs 4–6. In the dialectic analysis tree, the node represents argument proposed by agent in the argumentation and the arrow indicates the attack relation between arguments.

From the above process, we can draw the conclusion as follows:

1. *Agent*_1_ wins the game, and this case should be classified as “special priority”;
2. Finding the winning rule which can be seen as the high-quality knowledge: {critical_nursery, critical_housing, inconvenient_finance, priory_health → spec_prior; 100%; ∅; ∅;not_recom_health}

Through the above argumentation process, our model can evaluate and screen individual classification knowledge. At the end of argumentation, we can obtain a valuable winning rule which will be stored in the global knowledge base.

### Knowledge accumulation process of AMAJL

Though the above argumentation process, we have described how to extract high quality knowledge for classification. In this section, we design an experiment to demonstrate the knowledge accumulation process in the global knowledge base of ensemble classifier.

In this experiment, topics in training dataset are input into Arena continually until the global knowledge base converges. Here we define one group as 10 training cases. The convergence condition is that the rule number of global knowledge base does not increase with continuous five groups of training data.

**Fig 7:**
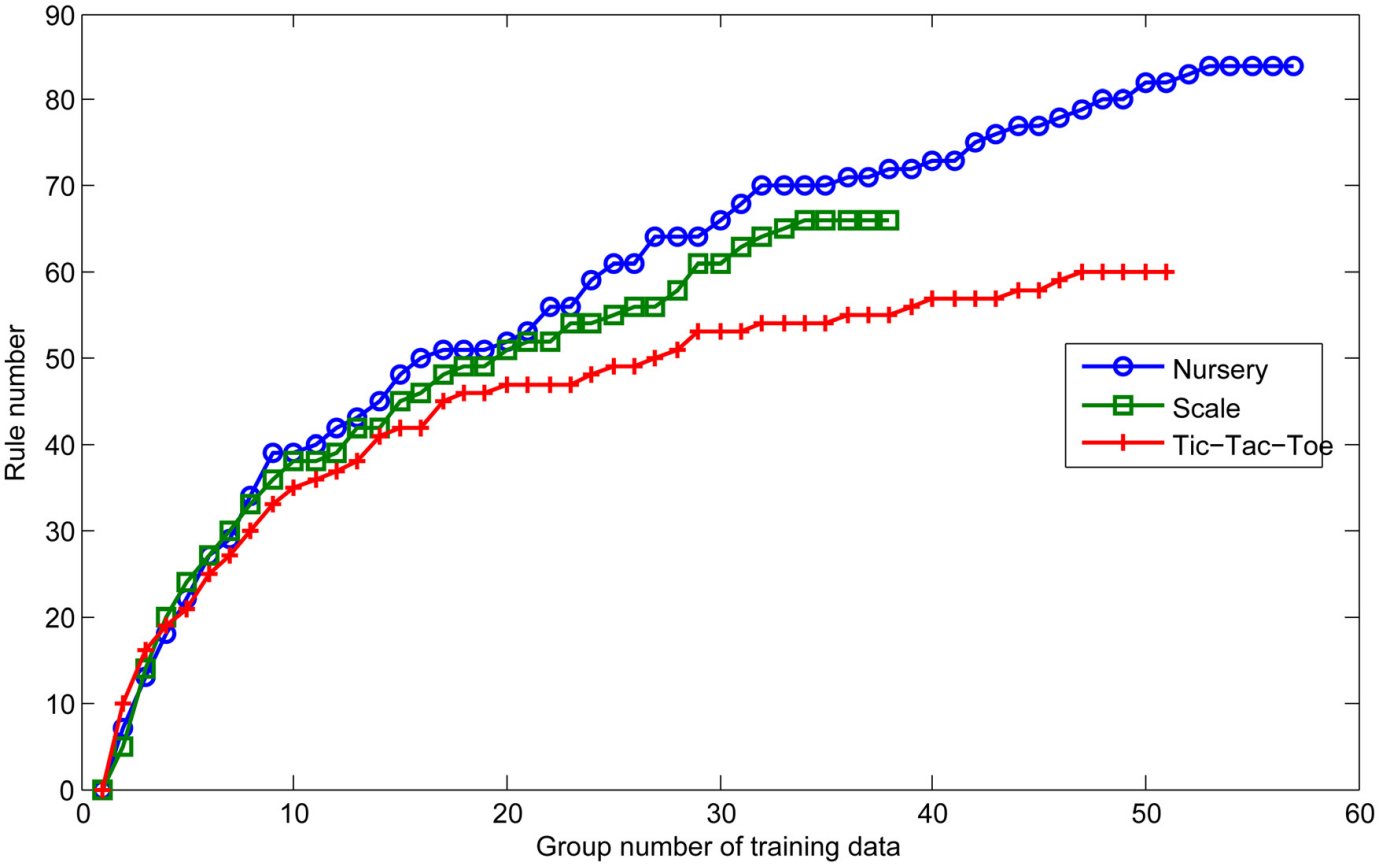
Knowledge accumulation process of AMAJL.

Fig 7 shows the knowledge accumulation process of AMAJL in three data sets. Can be seen from the figure, at the beginning of training process, rule number of the global knowledge base is growing rapidly, and have soon reached about 50 when we input 20 groups of training data. After that, rule numbers grow slowly, and only increase 21, 15 and 10 in three datasets respectively when training data reach 40 groups.

Then rules keep accumulating and eventually reach the convergent state. For the three datasets, rule numbers of the global knowledge base converge to 84, 66 and 60 respectively.

Experimental results show that, AMAJL method can effectively accumulate the high quality knowledge and the global knowledge base of ensemble classifier can converge in the three datasets.

### Classification performance

Through the above process of knowledge accumulation, we have obtained the ensemble classifier which can be directly applied in classification. Here we present an experimental analysis of our method. We address the task of classifying the unknown cases from the test set on several public datasets, and compare the results with the baseline methods.

First, we use following baseline methods to perform classification on the datasets, the results are reported in Table 4. As can be seen, classifiers have different performance on the given datasets. On average, KNN, SVM and RandomForest perform better than other classifiers.

**Table 4:** Accuracy of baseline methods.

Accuracy (%)	TFPC	NativeBayes	KNN-1	KNN-3	KNN-5	CART	SVM-R	SVM-L	RandomForest
Breast-Cancer	70.81	74.01	74.37	75.81	75.81	72.20	73.65	71.84	72.20
Lymph	65.52	85.14	80.41	81.08	79.73	81.08	79.73	81.76	84.46
Voting	94.82	90.95	91.38	92.24	92.67	96.98	96.98	96.98	96.12
Spect Heart	59.54	68.54	70.04	70.41	71.16	73.03	73.03	73.03	70.79
Nursery	77.77	90.32	98.38	98.38	98.38	99.56	97.62	93.17	98.98
Scale	76.64	91.36	83.84	83.84	83.84	78.24	91.04	90.72	77.44
Tic-Tac-Toe	67.27	69.46	98.85	98.85	98.85	92.99	88.60	98.54	96.65

It should be noted that TFPC, the association rule based classifier, has a relatively low classification performance. This is because TFPC just screen the association rules by pruning, rather than evaluating the quality of rules. Therefore, it cannot find high quality association rules for classification. To solve this problem, by using argumentation to integrate base classifiers, AMAJL can effectively extract high quality rules, which has significant advantages with respect to traditional ensemble methods.

The TCV test results of AMAJL on the seven datasets are shown in Table 5.

**Table 5:** TCV test results of AMAJL.

TCV test	Breast-Cancer	Lymph	Voting	Spect Heart	Nursery	Scale	Tic-Tac-Toe
Accuracy	74.07%	80.00%	97.83%	68.46%	91.14%	79.35%	91.05%
Variance	0.85%	0.88%	0.09%	0.26%	0.00%	0.19%	0.10%

In order to illustrate that AMAJL can effectively integrate base classifiers, we compare the results with Bagging and AdaBoost whose base classifiers choose CBA algorithm (Classification Based on Associations) in Tables 6 and 7.

**Table 6:** Accuracy of association rule based classifiers.

Accuracy (%)	AMAJL	Bagging	AdaBoost	TFPC
Breast-Cancer	74.07	73.29	69.31	70.81
Lymph	80.00	70.95	77.70	65.52
Voting	97.83	95.69	95.69	94.82
Spect Heart	68.46	65.92	69.29	59.54
Nursery	91.14	89.6	93.4	77.77
Scale	79.35	81.6	80.48	76.64
Tic-Tac-Toe	91.05	77.09	78.3	67.27

**Table 7:** Rule number of association rule based classifiers.

RuleNum	AMAJL	Bagging	AdaBoost	TFPC
Breast-Cancer	47.5	23.25	24	29
Lymph	12.5	8.5	6.5	12
Voting	9.5	6.5	3.25	16
Spect Heart	31.8	13	11.75	6.1
Nursery	100	226.25	202.5	40.6
Scale	61.3	67	55.75	18.7
Tic-Tac-Toe	64.2	105.5	110.5	74.4

In contrast with centralized classification algorithm TFPC, our method can outperform TFPC on all the seven datasets. The accuracy of AMAJL is higher than TFPC from a low of 2.71%, to a high of 23.78%. Bagging and AdaBoost methods are both superior to TFPC. Classification accuracy of TFPC is relatively low since TFPC method only uses single classifier for classification. AMAJL can combine multiple classifiers to get the high classification accuracy.

In contrast with traditional ensemble methods, we can find that, AMAJL as a novel ensemble learning algorithm has more advantages than Bagging and AdaBoost methods. For example, in *Tic-Tac-Toe*, AMAJL can obtain accuracy of up to 91.05% while accuracy of Bagging and AdaBoost are lower than 80%. Accuracy of AMAJL is 13.9% and 12.7% higher than Bagging and AdaBoost respectively. Similarly, in *Breast-Cancer*, *Lymph* and *Voting*, AMAJL can also outperform Bagging and AdaBoost. And in *Spect Heart*, *Nursery* and *Scale*, classification accuracy of AMAJL are comparable with Bagging and AdaBoost.

Furthermore, it should be noticed that we should also focus on knowledge scale of classifier in addition to classification accuracy. From Table 7 we can see, rule number of AMAJL is much smaller than that of Bagging and AdaBoost in *Nursery* and *Tic-Tac-Toe*, where AMAJL can only use 100 and 64.2 rules which are approximately half of rules in Bagging and AdaBoost. While in *Breast-Cancer*, *Lymph*, *Voting* and *Spect Heart*, rule numbers of AMAJL are relatively higher than Bagging and AdaBoost.

This is because that in *Nursery*, *scale* and *Tic-Tac-Toe*, we set low support thresholds, thereby generating a large number of rules. Baseline methods only use pruning to control the number of rules, which is difficult to compress knowledge scale. While AMAJL uses argumentation to eliminate weaker rules and extract high quality rules, so rules in AMAJL can be better compressed. In resting datasets, we set high support thresholds, thereby generating fewer rules. Baseline methods remove a large number of valuable rules by pruning. Although rule numbers of baseline methods are lower, it is difficult to ensure classification performance.

As a conclusion, from the above experimental results, we can find that AMAJL can effectively extract high quality knowledge for ensemble classifier and ensure high accuracy in classification as well.

### Parameter discussion

In ensemble learning methods, classification performance of ensemble classifier depends on not only integration strategy but also the ability of base classifiers. As a result, base classifier generation in AMAJL has a crucial effect on the quality of ensemble classifier. In this section, we will put particular emphasis on the parameter setting of AMAJL in the process of base classifier generation.

In base classifier generation, AMAJL uses association rule mining algorithm to achieve the base classifier construction. The pre-set of support and confidence threshold parameter is essential for association rule mining. Different support and confidence thresholds will lead to different mining results. Therefore, support and confidence threshold will directly affect the quality of association rule mining, then further affecting the classification performance of ensemble classifier. However, the support and confidence threshold is often difficult to set accurately and in most cases users need to judge by experience.

**Fig 8:**
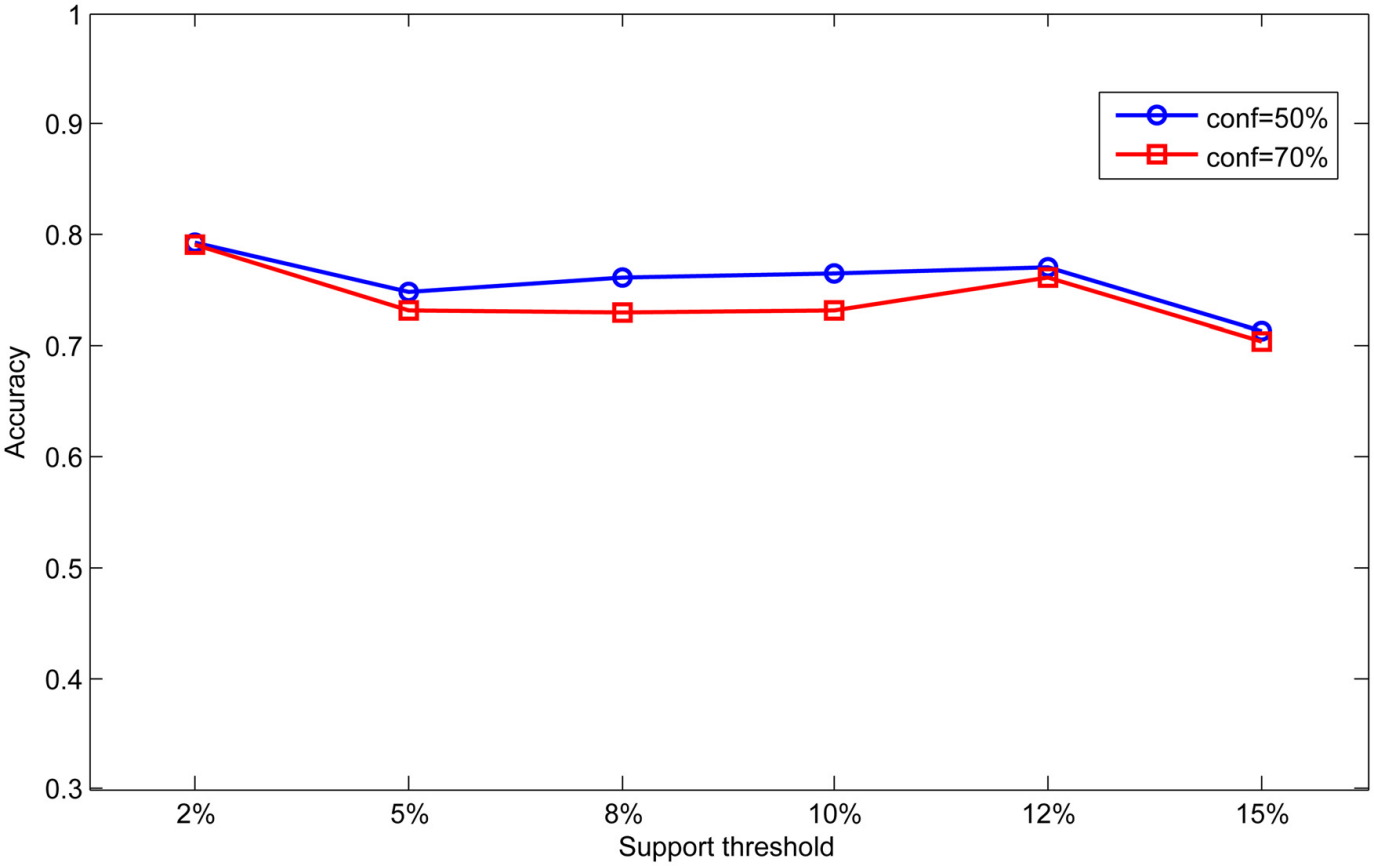
Effect of support on accuracy.

**Fig 9:**
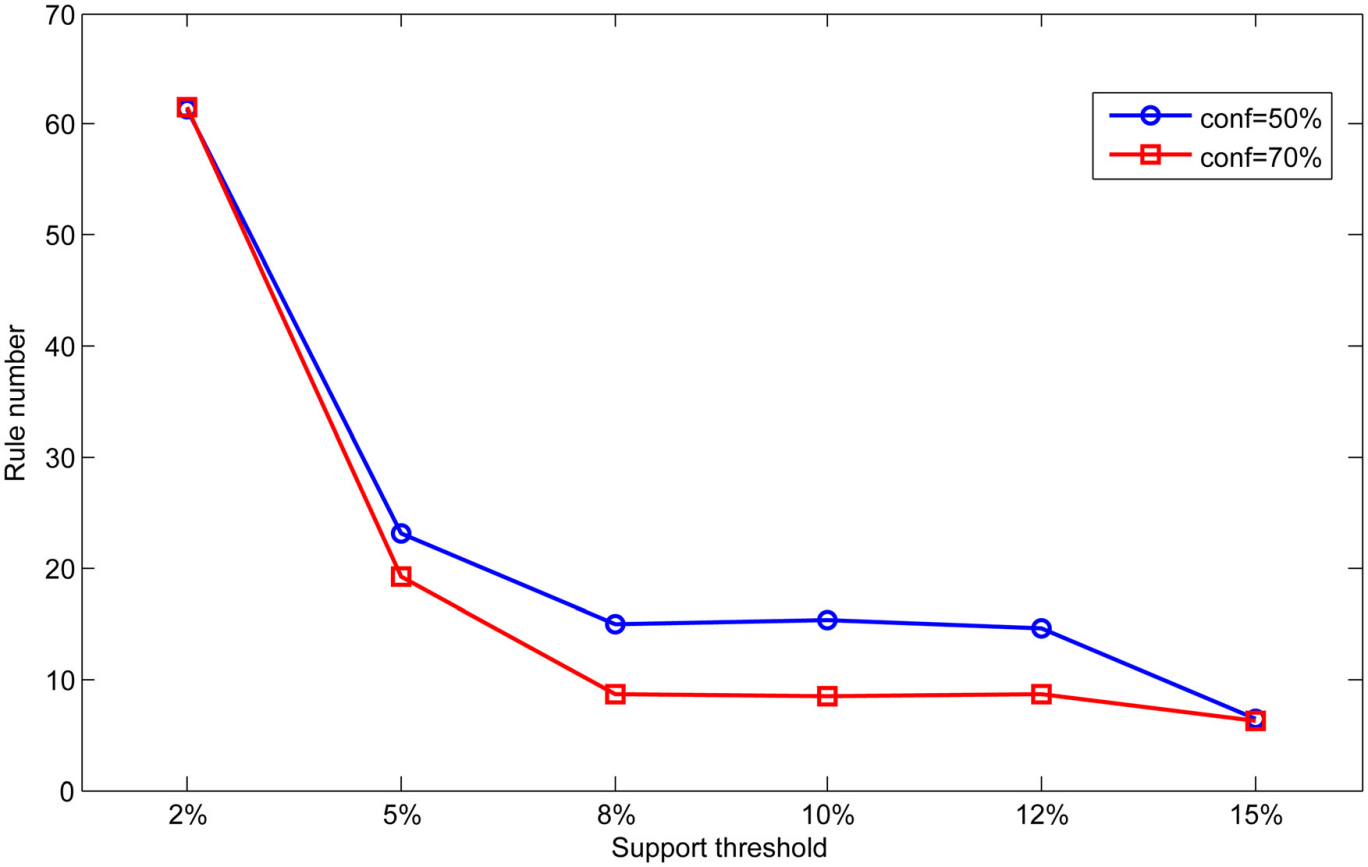
Effect of support on rule number.

**Fig 10:**
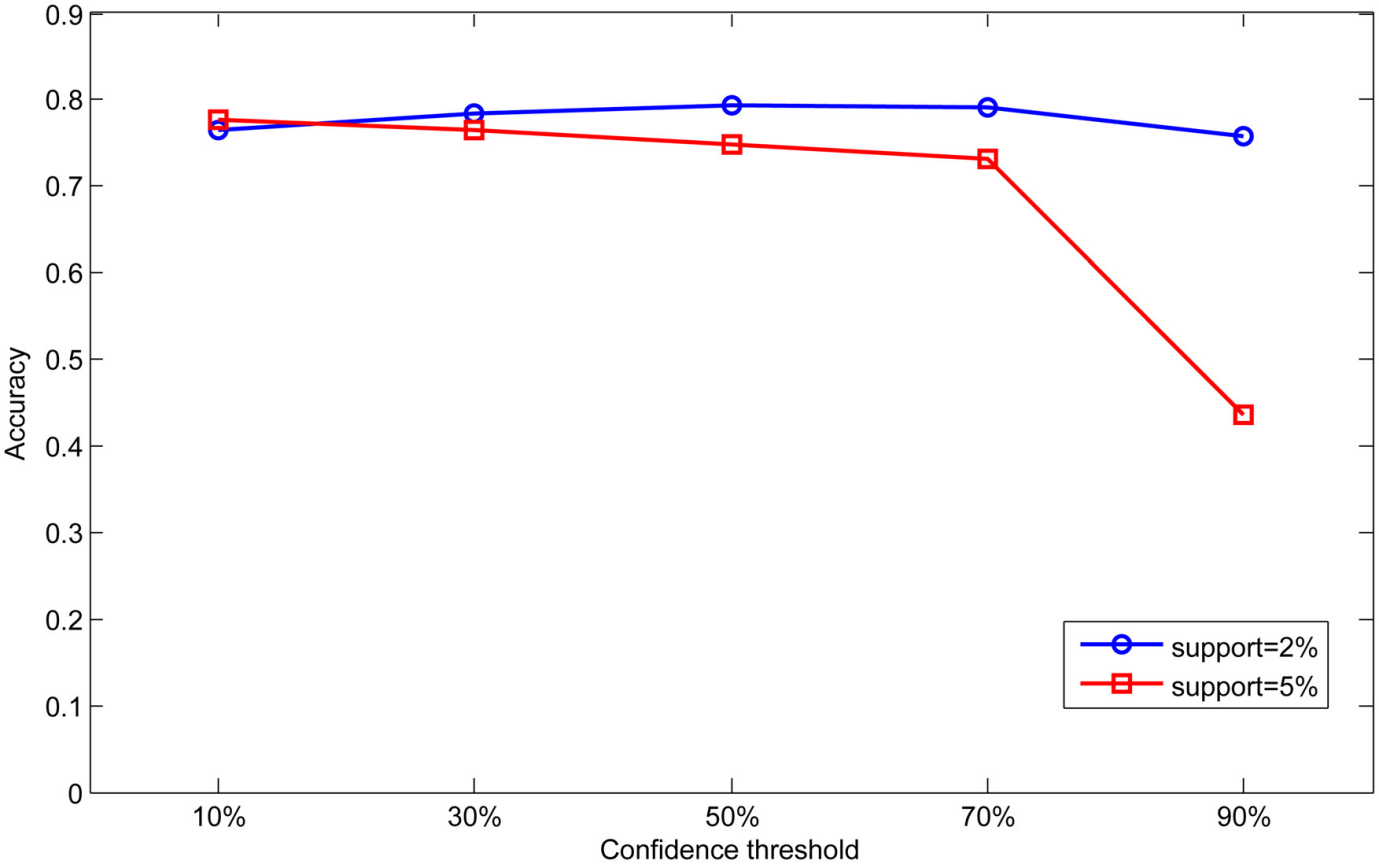
Effect of confidence on accuracy.

**Fig 11:**
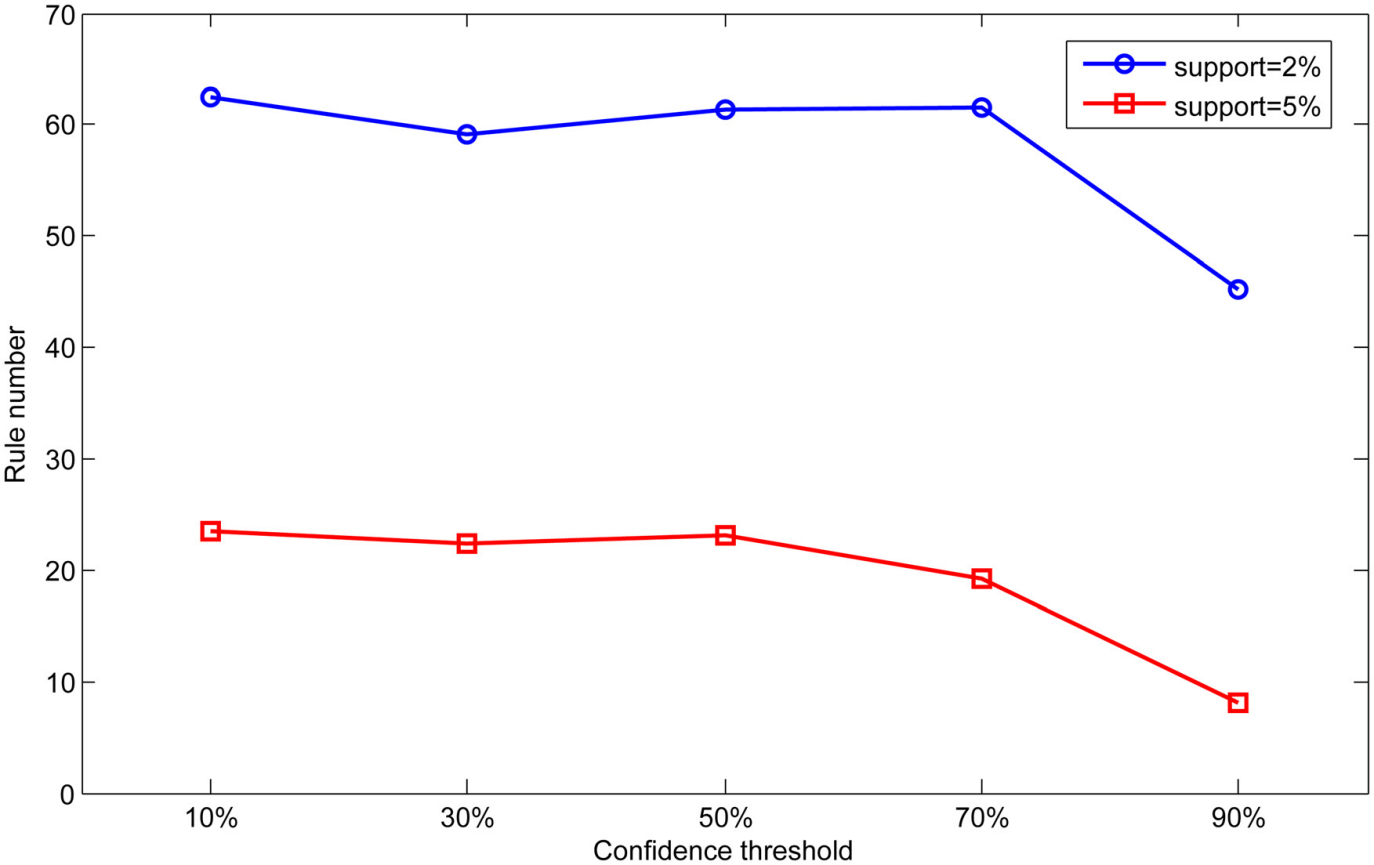
Effect of confidence on rule number.

In order to analyze the effect of different parameters, we set different support and confidence thresholds in scale dataset to analyze the influence of parameter on the classification results. First, we fix the confidence threshold as 50% and 70%, and vary the support threshold from 2% to 15% in this experiment. The results are shown in Figs 8 and 9. Then we fix the support threshold as 2% and 5%, and vary the value of confidence threshold from 10% to 90%. The results are shown in Figs 10 and 11.

As can be seen from Figs 8 and 9, accuracy and rule number at two confidence thresholds have the same trend as support threshold varies. With the increase of support threshold, accuracy drops slightly, while rule number has significantly decreased. In Figs 10 and 11, we can find that with the increase of confidence threshold, accuracy and number of rules do not change obviously at the beginning. However, when the confidence threshold continues to increase (higher than 50%), accuracy and rule number begin to decrease significantly.

Seen from the above experimental results, we can conclude that accuracy and rule number are sensitive to change of support and confidence threshold. This is because that support and confidence thresholds are used to filter rules in association rule mining process. Higher thresholds mean more strict measures to mine association rules, so rules in base classifiers will be much fewer for integration. As a result, knowledge in the global knowledge base will become scarce and limit the ability of ensemble classifier. In contrast, base classifiers can obtain more rules at lower thresholds resulting in better classification performance.

## Conclusions

In order to generate high performance ensemble classifier, we propose a novel joint learning method based on argumentation, AMAJL. In this method, argumentation is used as a novel ensemble strategy for knowledge integration, which is more logical and explicable than traditional ensemble method (such as voting). Meanwhile, by using argumentation, high-quality individual knowledge can be extracted to generate the independent ensemble classifier, which can be directly applied for classification without base classifiers.

Experiments show that, as a novel ensemble method, AMAJL outperforms the centralized classification method (TFPC) significantly. Meanwhile, compared to traditional ensemble methods, AMAJL can effectively extract high quality knowledge for ensemble classifier and ensure high classification accuracy as well, which indicates that argumentation as a novel ensemble strategy can improve the capability of knowledge integration effectively.

In our future research, we will further deepen the argumentation mechanism on joint learning method with bipolar argumentation model which will involve support behavior on the basis of existing attack behavior. Furthermore we will also try to apply other data mining methods such as decision tree to generate base classifiers in order to evaluate the universality of AMAJL.

## References

[1] Dietterich TG. In: Ensemble methods in machine learning. Springer; 2000 p. 1–15.

[2] Kuncheva LI. Combining pattern classifiers: methods and algorithms. John Wiley & Sons; 2004.

[3] Oza NC, Tumer K. Classifier ensembles: Select real-world applications. Information Fusion. 2008;9(1):4–20. 10.1016/j.inffus.2007.07.002

[4] Silva C, Lotric U, Ribeiro B, Dobnikar A. Distributed text classification with an ensemble kernel-based learning approach. Systems, Man, and Cybernetics, Part C: Applications and Reviews, IEEE Transactions on. 2010;40(3):287–297. 10.1109/TSMCC.2009.2038280

[5] Yang Y, Chen K. Time series clustering via RPCL network ensemble with different representations. Systems, Man, and Cybernetics, Part C: Applications and Reviews, IEEE Transactions on. 2011;41(2):190–199. 10.1109/TSMCC.2010.2052608

[6] Xu Y, Cao X, Qiao H. An efficient tree classifier ensemble-based approach for pedestrian detection. Systems, Man, and Cybernetics, Part B: Cybernetics, IEEE Transactions on. 2011;41(1):107–117. 10.1109/TSMCB.2010.2046890 20457550

[7] Polikar R. Ensemble based systems in decision making. Circuits and Systems Magazine, IEEE. 2006;6(3):21–45. 10.1109/MCAS.2006.1688199

[8] Rokach L. Ensemble-based classifiers. Artificial Intelligence Review. 2010;33(1–2):1–39. 10.1007/s10462-009-9124-7

[9] Breiman L. Bagging predictors. Machine learning. 1996;24(2):123–140. 10.1023/A:1018054314350

[10] Schapire RE. In: The boosting approach to machine learning: An overview. Springer; 2003 p. 149–171.

[11] Breiman L. Random forests. Machine learning. 2001;45(1):5–32. 10.1023/A:1010933404324

[12] Brown G. In: Ensemble learning. Springer; 2010 p. 312–320.

[13] Wardeh M, Bench-Capon T, Coenen F. PADUA: a protocol for argumentation dialogue using association rules. Artificial Intelligence and Law. 2009;17(3):183–215. 10.1007/s10506-009-9078-8

[14] Wardeh M, Bench-Capon T, Coenen F. In: Multi-party argument from experience. Springer; 2010 p. 216–235.

[15] Wardeh M, Bench-Capon T, Coenen F. Arguing from experience using multiple groups of agents. Argument and Computation. 2011;2(1):51–76. 10.1080/19462166.2010.528176

[16] Wardeh M, Coenen F, Bench-Capon T. Multi-agent based classification using argumentation from experience. Autonomous Agents and Multi-Agent Systems. 2012;25(3):447–474. 10.1007/s10458-012-9197-6

[17] Governatori G, Stranieri A. Towards the application of association rules for defeasible rules discovery. In: Jurix 2001;. p. 63–75.

[18] Možina M, Žabkar J, Bench-Capon T, Bratko I. Argument based machine learning applied to law. Artificial Intelligence and Law. 2005;13(1):53–73. 10.1007/s10506-006-9002-4

[19] Gómez, SA, Chesnevar, CI. Integrating defeasible argumentation and machine learning techniques. arXiv preprint cs/0402057. 2004.

[20] Ontanón S, Plaza E. In: Arguments and counterexamples in case-based joint deliberation. Springer; 2007 p. 36–53.

[21] Ontanón S, Plaza E. Multiagent inductive learning: an argumentation-based approach. In: Proceedings of the 27th International Conference on Machine Learning (ICML-10);. 2010. p. 839–846.

[22] Ontanón S, Plaza E. Coordinated inductive learning using argumentation-based communication. Autonomous Agents and Multi-Agent Systems. 2014;p. 1–39.

[23] Monteserin A, Amandi A. A reinforcement learning approach to improve the argument selection effectiveness in argumentation-based negotiation. Expert Systems with Applications. 2013;40(6):2182–2188. 10.1016/j.eswa.2012.10.045

[24] Gao Y, Toni F. In: Argumentation Accelerated Reinforcement Learning for RoboCup Keepaway-Takeaway. Springer; 2014 p. 79–94.

[25] Parikh D, Polikar R. An ensemble-based incremental learning approach to data fusion. Systems, Man, and Cybernetics, Part B: Cybernetics, IEEE Transactions on. 2007;37(2):437–450. 10.1109/TSMCB.2006.883873 17416170

[26] Zhou ZH. Ensemble methods: foundations and algorithms. CRC Press; 2012.

[27] Ho TK. The random subspace method for constructing decision forests. Pattern Analysis and Machine Intelligence, IEEE Transactions on. 1998;20(8):832–844. 10.1109/34.709601

[28] Rodriguez JJ, Kuncheva LI, Alonso CJ. Rotation forest: A new classifier ensemble method. Pattern Analysis and Machine Intelligence, IEEE Transactions on. 2006;28(10):1619–1630. 10.1109/TPAMI.2006.211 16986543

[29] Kuncheva LI. Using diversity measures for generating error-correcting output codes in classifier ensembles. Pattern Recognition Letters. 2005;26(1):83–90. 10.1016/j.patrec.2004.08.019

[30] Schapire RE. The strength of weak learnability. Machine learning. 1990;5(2):197–227. 10.1007/BF00116037

[31] Melville P, Mooney RJ. Creating diversity in ensembles using artificial data. Information Fusion. 2005;6(1):99–111. 10.1016/j.inffus.2004.04.001

[32] Brown, G. Diversity in neural network ensembles. 2004.

[33] Brown G, Wyatt J, Harris R, Yao X. Diversity creation methods: a survey and categorisation. Information Fusion. 2005;6(1):5–20. 10.1016/j.inffus.2004.04.004

[34] Galar M, Fernandez A, Barrenechea E, Bustince H, Herrera F. A review on ensembles for the class imbalance problem: bagging-, boosting-, and hybrid-based approaches. Systems, Man, and Cybernetics, Part C: Applications and Reviews, IEEE Transactions on. 2012;42(4):463–484. 10.1109/TSMCC.2011.2161285

[35] Chen P, Zhang D. In: Constructing Support Vector Machines Ensemble Classification Method for Imbalanced Datasets Based on Fuzzy Integral. Springer; 2014 p. 70–76.

[36] Coenen F, Leng P, Ahmed S. Data structure for association rule mining: T-trees and P-trees. IEEE Transactions on Knowledge and Data Engineering. 2004;16(6):774–778. 10.1109/TKDE.2004.8

[37] Nonaka I, Takeuchi H. The knowledge-creating company: How Japanese companies create the dynamics of innovation. Oxford university press; 1995.

[38] Skurichina M, Duin RP. Bagging for linear classifiers. Pattern Recognition. 1998;31(7):909–930. 10.1016/S0031-3203(97)00110-6

[39] Skurichina M, Duin RP. In: Bagging and the random subspace method for redundant feature spaces. Springer; 2001 p. 1–10.

[40] Freund Y, Schapire RE. A desicion-theoretic generalization of on-line learning and an application to boosting In: Computational learning theory. Springer; 1995 p. 23–37.

[41] Freund Y, Schapire RE. Experiments with a new boosting algorithm. In: ICML. vol. 96; 1996 p. 148–156.

[42] Coenen F, Leng P, Zhang L. In: Threshold tuning for improved classification association rule mining. Springer; 2005 p. 216–225.

[43] Chang CC, Lin CJ. LIBSVM: a library for support vector machines. ACM Transactions on Intelligent Systems and Technology (TIST). 2011;2(3):27.

[44] John GH, Langley P. Estimating continuous distributions in Bayesian classifiers In: Proceedings of the Eleventh conference on Uncertainty in artificial intelligence. Morgan Kaufmann Publishers Inc; 1995 p. 338–345.

[45] Breiman L, Friedman J, Stone CJ, Olshen RA. Classification and regression trees. CRC press; 1984.

[46] Aha DW, Kibler D, Albert MK. Instance-based learning algorithms. Machine learning. 1991;6(1):37–66. 10.1023/A:1022689900470

[47] Hall M, Frank E, Holmes G, Pfahringer B, Reutemann P, Witten IH. The WEKA data mining software: an update. ACM SIGKDD explorations newsletter. 2009;11(1):10–18. 10.1145/1656274.1656278

[48] Liu B, Hsu W, Ma Y. Integrating classification and association rule mining. In: Proceedings of the 4th ACM SIGKDD international conference on Knowledge discovery and data mining; 1998.

